# Balanced anesthesia in pigeons (*Columba livia*): a protocol that ensures stable vital parameters and feasibility during long surgeries in cognitive neuroscience

**DOI:** 10.3389/fphys.2024.1437890

**Published:** 2024-08-01

**Authors:** A. Serir, J. M. Tuff, N. Rook, E. Fongaro, T. Schreiber, E. Peus, O. Güntürkün, D. Manahan-Vaughan, J. Rose, R. Pusch

**Affiliations:** ^1^ Department of Biopsychology, Institute of Cognitive Neuroscience, Faculty of Psychology, Ruhr University Bochum, Bochum, Germany; ^2^ Department of Neurophysiology, Institute of Physiology, Medical Faculty, Ruhr University Bochum, Bochum, Germany; ^3^ Department of Anesthesiology, Center for Anesthesiology and Intensive Care Medicine, University Medical Center Hamburg-Eppendorf, Hamburg, Germany; ^4^ Max Planck School of Cognition, Leipzig, Germany; ^5^ Department of Neural Basis of Learning, Institute of Cognitive Neuroscience, Faculty of Psychology, Ruhr University Bochum, Bochum, Germany; ^6^ Pigeon Clinic Essen, Essen, Germany

**Keywords:** cognitive neuroscience, isoflurane, ketamine-xylazine, avian model, avian anesthesia, surgery protocol, pigeon vital parameters

## Abstract

In neuroscience, numerous experimental procedures in animal models require surgical interventions, such as the implantation of recording electrodes or cannulas before main experiments. These surgeries can take several hours and should rely on principles that are common in the field of research and medicine. Considering the characteristics of the avian respiratory physiology, the development of a safe and replicable protocol for birds is necessary to minimize side effects of anesthetic agents, circumvent technical limitations due to the insufficient availability of patient monitoring, and to maintain stable intraoperative anesthesia. Through the consistent and responsible implementation of the three R principle of animal welfare in science (“Replace, Reduce, Refine”), we aimed to optimize experimental methods to minimize the burden on pigeons (*Columba livia*) during surgical procedures. Here, surgeries were conducted under balanced anesthesia and perioperative monitoring of heart rate, oxygen saturation, body temperature, and the reflex state. The protocol we developed is based on the combination of injectable and inhalative anesthetic drugs [ketamine, xylazine, and isoflurane, supported by the application of an opiate for analgesia (e.g., butorphanol, buprenorphine)]. The combination of ketamine and xylazine with a pain killer is established in veterinary medicine across a vast variety of species. Practicability was verified by survival of the animals, fast and smooth recovery quantified by clinical examination, sufficiency, and stability of anesthesia. Independent of painful stimuli like incision or drilling, or duration of surgery, vital parameters were within known physiological ranges for pigeons. Our approach provides a safe and conservative protocol for surgeries of extended duration for scientific applications as well as for veterinary medicine in pigeons which can be adapted to other bird species.

## Introduction

In life sciences, such as biology and medicine, animal models are often used to study physiological processes and develop novel products or new therapeutic strategies (Gargiulo et al., 2012). While in some research areas animal models can be substituted by cell lines, the field of cognitive neuroscience depends on animal models, since cognition cannot be investigated *ex vivo*. Several techniques used to investigate the neuronal correlates of cognition such as anatomical studies (Hubel and Wiesel, 1962), *in vivo* electrophysiology (Azizi et al., 2019), functional magnetic resonance imaging [fMRI (Groof et al., 2013; Behroozi et al., 2020)], optogenetics (Deisseroth, 2011; Rook et al., 2021a) calcium imaging (Dana et al., 2019) and tracing for anatomic studies (Rook et al., 2023; Steinemer et al., 2024) require intracranial injections or complex implantations, which can demand extended durations of surgery and anesthesia. Advancements in anesthetic techniques and devices that control vital signs allow these surgical procedures to take place at the standard of veterinary medicine. Anesthesia, in all its dimensions, is important to ensure significant pain reduction, including complete eradication of perception, periprocedural pain, and immobilization purposes to obtain accurate and safe procedures for the animal (Graham and Prescott, 2015; Seok et al., 2016). The analgesic and hypnotic components of anesthesia minimize animal stress and suffering (Gargiulo et al., 2012). However, anesthesia can also result in long-lasting cognitive deficits such as impaired learning and memory, especially when animals are relatively young as shown for rodents and primate species (Jevtovic-Todorovic et al., 2003; Lu et al., 2006; Paule et al., 2011). Thus, especially in cognitive neuroscience, such side effects caused by surgical interventions and anesthesia need to be avoided to prevent neurological deficits and ensure unimpaired cognitive capacities.

Apart from widely used mammalian model organisms, birds are prominent models in cognitive neuroscience. For example, homing pigeons (*Columba livia*) serve as a classical model in psychology to study the neurobiological fundaments of learning and memory (Güntürkün et al., 2014; Rook et al., 2021b; Packheiser et al., 2021; Pusch et al., 2023). Much of the technology developed for small animal (rodents, cat and dog species) anesthesia has been adapted for use in birds, including important advances in patient monitoring (Degernes, 2008). However, because avian anatomy and physiology vary considerably from mammalian species, there are limitations to the use of these technologies in avian anesthesia (Lierz and Korbel, 2012). Bird anesthesia is challenging for several reasons including the animals' size, metabolic rate, breathing physiology, and unknown sensitivity to drugs that are well-established for mammals, including humans (Lierz and Korbel, 2012).

A variety of injectable and inhalative anesthetic drugs have been documented in widely diverse avian species for veterinary protocols (O'Kane et al., 2016; Paul-Murphy and Fialkowski, 2001). One of the most commonly used injectable drugs is ketamine. Ketamine is a non-competitive antagonist of the ionotropic N-methyl-D-aspartate (NMDA) receptor (Orser et al., 1997). It also modulates and activates several subtypes of the gamma-Aminobutyric acid A (GABA_A_) receptor in contrast to other NMDAR-antagonists, such as phencyclidine and dizocilpine (Hevers et al., 2008). A weak pharmacological agonism towards opiate receptors has been described in the literature (Finck and Ngai, 1982). A characteristic effect of medication with ketamine is dissociative anesthesia, which is defined as anesthesia with the persistence of basal reflexes and the absence of cardiac depression (Anis et al., 1983; Morris et al., 2009; Trimmel et al., 2018). Amnesia caused by the NMDAR-antagonism and potent analgesia because of the interaction of earlier described mechanisms (NMDAR-antagonism and GABA and opiate receptor-agonism) are key features of ketamine anesthesia (Anis et al., 1983). It stimulates cardiovascular functions and increases heart rate and mean arterial blood pressure (Orser et al., 1997). When used in monotherapy, it can induce muscular hypertonicity, convulsions, and other adverse effects like postoperative amnesia. Especially the latter point poses a potential problem for cognitive neuroscience.

To minimize these effects, ketamine is commonly administered in combination with benzodiazepines or alpha-2 agonists such as xylazine (Maiti et al., 2006; Muir et al., 1977; Özkan et al., 2010). Xylazine is an agonist of the alpha-2 adrenergic receptors in the central nervous system (CNS). Xylazine attenuates the neurotoxic effects of other general anesthetics induced by, e.g., isoflurane (Greene and Thurmon, 1988; Sanders et al., 2009). It has sedative, analgesic and muscle relaxing effects which made xylazine, especially in conjunction with ketamine, a well-established drug in veterinary medicine (Muir et al., 1977; Haskins et al., 1986; Atalan et al., 2002). The main side effects are bradycardia, respiratory depression, vagus nerve stimulation, and arrhythmias (Greene and Thurmon, 1988). Dimensions of mentioned side effects vary significantly between species (Muir et al., 1977; Haskins et al., 1986; Aithal et al., 1997).

However, in avian veterinary medicine, volatile anesthetics are used more widely than intravenous anesthesia (Botman et al., 2016b), given that inhaled agents have numerous advantages compared to injectable anesthetics in birds. Inhalative anesthetic agents, such as isoflurane and sevoflurane can be titrated to effect, provide fast and smooth induction of anesthesia as well as fast recovery (Miller and Buttrick, 1999; Botman et al., 2016b). Isoflurane is a volatile anesthetic of the flurane-subgroup, which has been used for general anesthesia since the pioneer days of modern surgery (Tonner, 2005). In contrast to intravenous anesthetics, the exact mechanism underlying isoflurane anesthesia or other volatiles remains poorly understood. Different effects on the GABA-, glutamate-, and glycine-receptors are observed (Jones et al., 1992; Jenkins et al., 1999). However, how the interaction of these effects causes general anesthesia remains a target of further research (Ou et al., 2020).

Despite the analgesic effects of the abovementioned drugs, additional pain reduction is required and can be achieved using opioids such as Butorphanol. Butorphanol is a painkiller from the chemical group of opiates and is an agonist on the κ- and δ-opiate receptors and an antagonist on the µ-opiate receptor. The affinity to the receptors is described as 1:4:25 (µ, δ, κ) (Commiskey et al., 2005). Regarding respiratory depression, butorphanol shows a ceiling effect and can be antagonized with naloxone (Rosow, 1988). This makes butorphanol a commonly used opiate analgesic in veterinary medicine (Schatzmann et al., 2001; Grimm et al., 2000; Lierz and Korbel, 2012).

In human medicine, it has become common practice not just to use a single anesthetic drug, but to induce anesthesia using multiple components, as part of a balanced anesthesia strategy. Balanced anesthesia lacks a clear-cut definition but integrates the use of volatile anesthetics and intravenous components aiming for synergistic effects of any component and minimizing side effects due to drastic dose reduction for each component (Brown et al., 2018). It is an equivalent alternative to total intravenous anesthesia (TIVA) (Fitzgerald and Cooper, 1990; Tonner, 2005) and is even preferred for patients with pulmonary diseases, which can lead to hyperreactive respiratory organs and demanding respiratory physiology (Goff et al., 2000). Therefore, the unique challenges concerning the respiratory physiology of avian species and the current evidence suggest potential benefits from balanced anesthesia and is therefore also one of the main objectives of this study.

During surgery and anesthesia, it is essential to track vital parameters to monitor the patient’s physiological condition and state of anesthesia. However, literature reporting vital signs for avian species is currently inconsistent. Some studies report heart rates for pigeons that exceed 200 beats per minute (bpm) (Lopez Murcia et al., 2005; Omobowale et al., 2017). Other studies report heart rates around 120–160 bpm (Calder, 1968; Botman et al., 2016a; Botman et al., 2016b; Behroozi et al., 2020), which are significantly different from previous studies on the one hand, but still provides a value that is plausible for animals of this size in a resting state, on the other hand. Heterogeneity concerning pigeons’ vital parameters in the awake state and its differences to anesthetized physiology results from a lack of information concerning the animal’s habituation to novel, restraining, ergo stressful measurement procedures (Omobowale et al., 2017; Varshney, 2017). Missing standardization of habituation to measurements may have led to inconsistent reports of birds’ heart rates and other vital parameters when animals are awake and behaving under the absence of stress as this is not reported in the studies reported beforehand. Furthermore, the dynamics of vital parameters have not been consistently tested for surgeries longer than 2–2.5 hours (h) (Lumeij and Deenik, 2003; Sabiza et al., 2012; Botman et al., 2016b).

This study therefore focused on establishing an effective protocol for safe anesthesia in birds, especially for prolonged surgeries. Used for this, we assessed the effects of balanced anesthesia (intravenous and inhalation anesthetic agents) in pigeons, inspired by the standard of care in human medicine. In our protocol, anesthesia was induced with a combination of ketamine and xylazine. Analgesia was enabled by the opiate butorphanol. Maintenance of the anesthesia depth was ensured by the volatile anesthetic, isoflurane. To investigate the perioperative course to identify aspects for further improvements, we monitored and recorded vital signs such as heart rate (Hr), oxygen saturation (SpO_2_), and temperature (Temp) under conditions of spontaneous breathing and supplementary oxygen supply (in case of anesthesia). To study conditions for sufficient analgesia under conditions of stereotactic surgery, nine surgeries of the same type (stereotactic electrode implantation for electrophysiology) were analyzed again at defined time intervals. Our approach set about to assess the effectivity of balanced anesthesia in avian research and indicated that this strategy serves to reduce the physiological burden and risk of prolonged anesthesia in pigeons.

## Materials and methods

### Animals

This study is based on the recordings from 30 adult homing pigeons (*Columba livia, n* = 21 for surgical recordings, *n* = 9 awake recordings), which were obtained from local breeders for behavioral and neuroscientific experiments that were approved in advance with a separate ethics application. Animals of both sexes have been used in our experiments. The weight of the animals ranged from 440 g to 533 g (mean = 481 g). Their age ranged from 3–10 years (mean = 5.2 years). Animals for awake recordings were completely naïve to handling and experiments beforehand to proper investigate habituation to handling. Before surgery, the animals were housed in an aviary measuring (156 cm × 128 cm × 180 cm) and in individual cages (45 cm × 40 cm × 35 cm) after surgery. Isolation was necessary due to the increased risk of injury in cases in which the pigeons received skull implants. The pigeons from the surgery group had *ad libitum* access to water and their weight was maintained at least approximately 85% of their free-feeding weight so that the animals have a certain reserve for the post-operative phase in which they are not initially fed. All procedures, containing handling, interventions, and measurements were performed regarding the principles of care and use of animals in science requested by German Animal Welfare Law to prevent cruelty towards animals and reduce suffering and the number of animals used. It is also congruent with the guidelines of the European Communities Council Directives of 22 September 2010 (2010/63/EU). Surgical procedures before the conduction of our experiments were in concordance with the animal welfare law and the three R’s of animal welfare (Replace, Reduce, Refine) and were approved in advance by the national ethics committee of the State of North Rhine-Westphalia (NRW), Germany (Landesamt für Arbeitschutz, Naturschutz, Umweltschutz und Verbraucherschutz, LANUV NRW; Application numbers: 81-02.04.2023.A292, 81-02.04.2021.A324, 81-02.04.2021.A300, and 81-02.04.2021.A240).

### Anesthesia protocol

**FIGURE 1:**
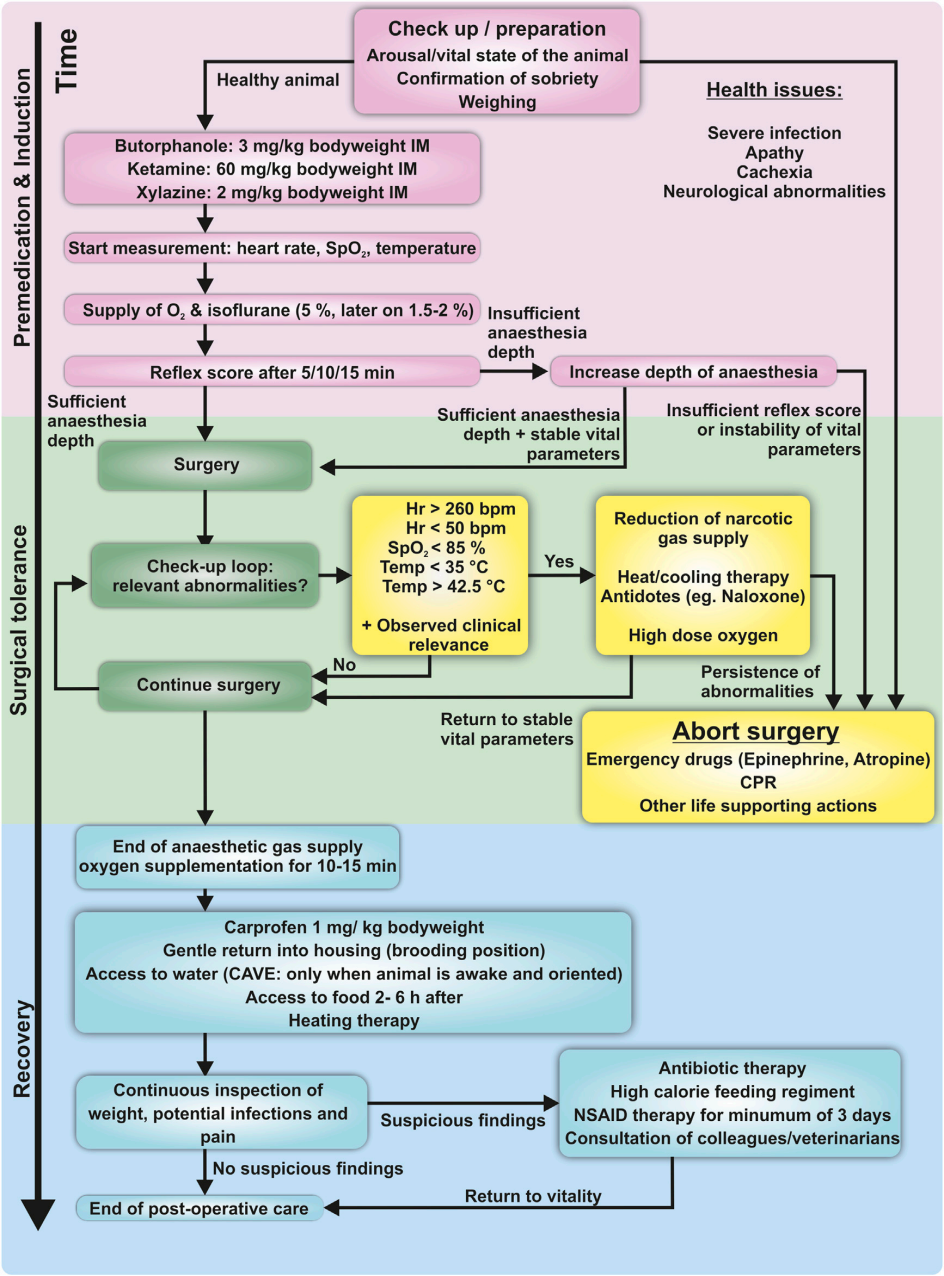
Schematic flowchart of the protocol used in this study, including physiological endpoints. Induction confirms the animal’s capability to undergo surgery. A calm, food-deprived and healthy animal is an obligatory condition. Induction of anesthesia is conducted via injection the pectoral muscle. After supply with oxygen and volatile anesthesia, reflexes are checked to verify the depth of anesthesia. During surgery, a check-up loop is used to identify emergencies. In case of abnormalities, immediate action is required to avoid suffering or life-threatening damage to the animal. Vital abnormalities which are not reversible should immediately lead to abortion of surgery. The surgeon/anesthesiologist has the responsibility to keep track of these changes for the total duration of the procedure. The recovery phase includes postoperative analgesia and supply with water and food once the animal is fully orientated. Hypothermia and postoperative shivering are avoided with heating blankets or infrared lamps.

The animals were food-deprived for 12 h before surgery to avoid perioperative aspiration of gastric and goitrous contents. Butorphanol (Dolorex^®^, MSD Animal Health, Germany, 3 mg/kg body weight intramuscular (IM)) or Buprenorphine (Buprenovet^®^, VetViva Richter GmbH, Austria, 0.5 mg/kg body weight IM) were injected into the pectoral muscle a few minutes before application of the anesthetic drugs for premedication leading into mild sedation. Anesthesia was induced with a mixture of ketamine and xylazine (Ketavet^®^, Zoetis Deutschland GmbH, Germany, 60 mg/kg body weight IM and Rompun^®^, Bayer AG, Germany, 2 mg/kg body weight IM) into the other chest muscle. Maintenance of anesthesia was ensured with isoflurane (Forene^®^, AbbVie AG, Switzerland) in conjunction with pure oxygen (3–5%-vol isoflurane fraction until surgical tolerance was achieved, 1.5%–2.0% for maintenance), flow rate of oxygen varied from 0.3 L/min to 0.5 L/min, depending on the balance of sufficient oxygenation and surgical tolerance as defined in the following. A score based on the examination of protective reflexes verified satisfactory depth of anesthesia. This score assesses vigilance, central eye-muscle reflexes, muscle tonicity, and pain reflexes (Supplementary Table 2). A maximum of 3–4 points is considered as surgical tolerance accompanied by the absence of vigilance and absence of physiological responses to painful stimuli (Lierz and Korbel, 2012; Erhardt et al., 2012). The pigeon was then placed on an adjustable heating blanket for temperature management. A non-rebreathing system was used for ventilatory support (Komesaroff Mark V, Medical development international limited, Australia). In this type of system, little or no exhaled gas is returned to the animal, rather it is exhaled through a valve into a scavenger hose. Preparations for emergencies consisted of the availability of emergency medication (e.g., atropine 0.5 mg/kg body weight IM, epinephrine 0.5 mg/kg body weight IM (Lierz and Korbel, 2012, etc.) and the regular evaluation of vital parameters. Experiment termination criteria were defined in concordance with current available literature and clinical expertise and consisted of comprised excessive bradycardia or tachycardia (Hr below 50 bpm or above 260 bpm), severe hypo- and hyperthermia (Temp below 35°C and above 42.5°C), and quantifiable hypoxemia (SpO_2_ ≤ 85%) (Hatt, 2002; Lopez Murcia et al., 2005; Lierz and Korbel, 2012; Botman et al., 2016a; Botman et al., 2016b; Omobowale et al., 2017). General complications during the course of anesthesia such as excessive bleeding, or other injuries due to the surgical procedure contribute to the abortion criteria as well. It is also important for the experimenter to determine the clinical relevance of these changes based on clinical examination of the animal. If abnormalities are unchangeable under general anesthesia, surgery should be aborted, and the animal should be allowed to wake up under oxygenation (Figure 1).

After surgery, the animal continued to be supplied with oxygen for 10–15 min and postoperative analgesia with a non-steroidal anti-inflammatory drug (NSAID; Rimadyl^®^, Pfizer Deutschland GmbH, Germany)/carprofen 1 mg/kg body weight IM was provided. A flowchart of the practical implementation of this protocol is shown in Figure 1.

### Surgical procedures and experimental recording

Following anesthesia, the pigeons underwent procedures relevant for post-operative research experimentation. These techniques cover surgeries for the implantation of electrodes for *in vivo* electrophysiology (Azizi et al., 2019) and cannulas for pharmacological injections (Gao et al., 2019), viral transfection and fiber optic implants for optogenetics (Rook et al., 2021a), and the fixation of head blocks for awake fMRI studies in pigeons (Behroozi et al., 2020). The procedures were all quite similar. After stereotactic fixation, the skin was incised, the cranium was drilled and the dura was incised. The implantation of the device then differed depending on the objective of the experiment.

A typical surgical procedure was performed as follows: a skin incision was made followed by exposure of the animal’s skull. Using a stereotactic atlas (Karten and Hodos, 1967), the coordinates for drilling craniotomies were determined. The bone fragment from the craniotomy was removed and the underlying dura mater was prepared and incised (Azizi et al., 2019; Gao et al., 2019; Rook et al., 2021a). Due to the nociceptive innervation of the dura (Lv et al., 2014), this time interval, at least containing ten minutes with drilling and dura incisions, was defined as the interval of potentially higher pain salience, and was used for further analysis of heart rates, sufficiency of analgesia and overall anesthesia quality. The implants for electrophysiological measurements were inserted into the brain tissue reaching a depth of several hundred micrometers to several millimeters. The vital parameters were measured with a multiparameter veterinary monitor (Lutech Datalys V, Lutech Veterinary Industries Inc., NY, United States).

Due to the initial hemodynamic situation and fluid saturation and expected low blood and liquid loss during brain surgeries compared to general surgery or cardiac surgery (Montroy et al., 2020), perioperative liquid infusion therapy was considered as not obligatory, but individually available if needed. Recordings started right after the injection of butorphanol, ketamine, and xylazine. When anesthesia reached a sufficient depth, which was verified by the evaluation of the reflex status inspired by previously examined anesthesia depth assessments (Erhardt et al., 2012; Lierz and Korbel, 2012), the animal was positioned in a stereotactic frame. Here, a complete loss of consciousness accompanied by a lack of reaction to targeted pain stimuli (pinching the interphalangeal skin) is mandatory. In addition, a maximum score of 3–4 points in our reflex score is interpreted as tolerance. Detailed assessment is explained in our supplementary material (Supplementary Table 2).

The last vital parameters (Hr, SpO_2_, Temp) were recorded after surgery when the animal was removed from the stereotactic apparatus and a 5 min interval of non-invasive spontaneous ventilation with fresh oxygen flow 0.3 L/min had elapsed. From now on, anesthesia became flatter, as the animal wakes up from surgery.

### Awake data recording

To obtain reliable references for the dynamics of vital parameters during surgery, we performed recordings of basic vital signs in a separate cohort of awake animals. The animals were gently restrained and placed in an experimental chamber with white noise to create an environment that provides low sensory stimulation. The eyes were covered with a blanket with a velcro fastener to further reduce stress. A leg was gently fixed with skin friendly tape to ensure permanent measurements of the oxygen saturation (SpO_2_, 0%–100%) and heart rate (Hr, measured in beats per minute (bpm)) with a pulse oximeter, applied on the metatarsophalangeal joints. The thermometer measured cloacal temperature (Temp, measured in °C) as a precise estimation of body core temperature. The cloaca of the animal was locally anesthetized with Xylocaine 2% (20 mg/g Lidocaine hydrochloride: AstraZeneca GmbH, Germany) beforehand. Recording sessions lasted for 30–45 min each. Measurements were conducted on three consecutive days at the same time in the morning to account for the influence of circadian rhythm (Oshima et al., 1989; Chen and Yang, 2015) and to reveal potential habituation effects to measuring and handling.

### Data analysis

Oxygen saturation, heart rate, and cloacal temperature for awake and anesthetized animals were recorded during this study as mentioned in the introduction in awake and anesthetized animals as well. Every minute of recording is represented as a data point for each of the three parameters. The recovery time was defined as the time between the end of the anesthetic gas supply and the first orientation reactions after awakening. We used the Kolmogorov-Smirnov test to test for normal distribution of the data and Levene’s test to test for the homogeneity of variances.

A one-way repeated measures ANOVA was calculated to compare heart rates, oxygen saturation, and cloacal temperature of awake animals measured over 3 days to investigate a potential stress reduction (within subject) effect (due to the animals being habituated to the procedure). Post-hoc tests for pairwise comparisons were Bonferroni corrected. Intraoperative heart rates and body temperatures were compared to the parameters of awake pigeons with *t*-tests. Intraoperative oxygen saturations were compared using a Mann-Whitney U test. *t*-test for dependent samples was used to evaluate differences in heart rates within surgeries due to the application of pain stimuli. For the investigation of time-dependent effects of the anesthetic protocol on heart rates and temperature between the ten shortest and the ten longest surgeries, a *t*-test was used. In case of oxygen saturation, a Mann-Whitney U test was applied to test for time-dependent anesthesia effects (IBM SPSS Version 25, IBM Corp, United States).

Vital parameters are shown as mean ± standard deviation (SD). For all comparisons, the significance level was set to alpha = 0.05.

## Results

This study was performed to provide a protocol for safe and effective anesthesia in pigeons. Therefore, vital parameters (heart rate, oxygen saturation, and temperature) and clinical outcomes of 21 adult homing pigeons that underwent surgeries for neuroscientific experiments were assessed. In one animal, anesthesia and surgery were discontinued due to hemodynamic instability and are therefore analyzed separately. Vital parameters of anesthetized animals were compared to those of nine awake pigeons. Vital signs were further evaluated regarding potential time-dependent effects of anesthesia, as well as during phases of noxious stimuli to verify the efficacy of anesthesia.

### Animal’s baseline heart rate in awake state is dependent on habituation to measurement

**FIGURE 2:**
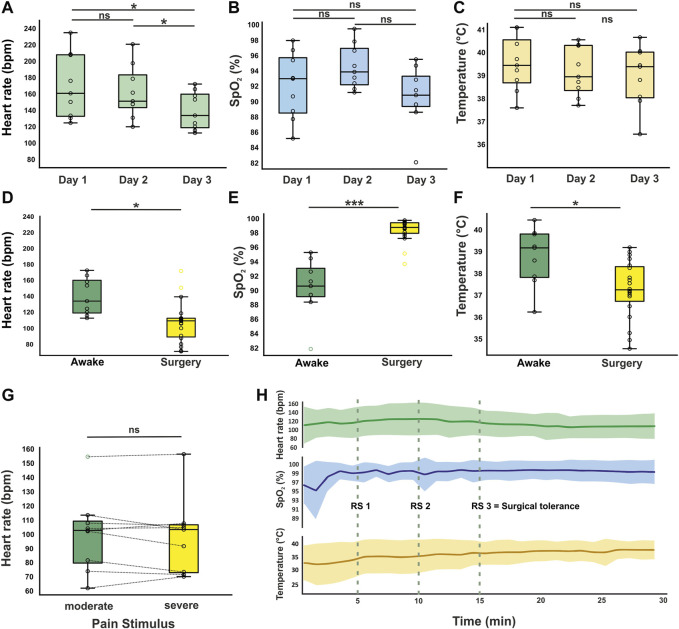
Vital parameters in awake and anesthetized animals. Heart rate **(A)**, oxygen saturation **(B)**, and cloacal temperature **(C)** of awake animals recorded on three consecutive days (*n* = 9). On day three, a significant decrease in heart rate was observed compared to day 1 and day 2. Oxygen saturation and body temperature did not vary significantly during habituation. **(D–F)** Vital parameters from the surgery group (*n* = 20) were compared to day 3 of awake recordings. **(G)** Mean heart rate ± SD during potential pain stimuli compared to time intervals with no noxious stimuli (*n* = 9 surgeries). No significant increase of heart rate was observed during pain stimuli. **(H)** Vital parameters trend for first 30 min of surgery (mean ± SD). At RS 1-3 the reflex state has been examined in every animal. The fluctuation in body temperature decreases with advancing depth of anesthesia (SD = 3.3°C during first 15 min and 1.6°C for second 15 min). SpO_2_ shows a ceiling effect due to the exposure of the animals to the narcotic gas combined with pure oxygen. */***Significant difference with *p* < 0.05/0.001. Boxplots represent the lower quartile (Q1), the median and the upper quartile (Q3). Whiskers represent Q1−1.5 * IQR and Q3 + 1.5 IQR.

Vital parameters during anesthesia were compared to reliable awake data to ensure the adequate classification of anesthetized measurements. Regarding the heterogeneity of current literature and the gap between human/mammalian data availability and avian pendants outlined by several cited studies with scattering vital parameter reporting, we measured vital signs in 9 awake animals in addition to 20 anesthetized animals. In awake recordings, the heart rates measured on three consecutive days differed significantly from each other (F_(2,16)_ = 12.978, *p* < 0.001). The mean heart rate on day three (139 bpm ± 22 SD) was significantly lower compared to day one (170 bpm ± 37 SD, *p* = 0.014) and day two (162 bpm ± 30 SD, *p* = 0.018) in conjunction with a reduced standard deviation, further indicating stability against environmental influences. There was no significant difference between the mean heart rate on day one and day two (*p* = 0.255, Figure 2A). For oxygen saturation, there were no significant differences between the three measured time points (F_(2, 16)_ = 2.521, *p* = 0.112; day one: 92% ± 4 SD, day two: 95% ± 3 SD, day three: 91% ± 4 SD, Figure 2B). Similarly, there was no significant effect of day on temperature (F_(2, 16)_ = 0.909, *p* = 0.423; day one: 39.5°C ± 1.1 SD, day two: 39.2°C ± 1.0 SD, day three: 39.0°C ± 1.2 SD, Figure 2C and Table 1).

**TABLE 1:** Vital parameters in awake animals (*n* = 9) over three consecutive days compared to anesthetized animals (*n* = 20, mean ± SD).

Condition	Heart rate (bpm)	SpO_2_ (%)	Temperature (°C)
Day 1	170 ± 37	92 ± 4	39.5 ± 1.2
Day 2	162 ± 30	95 ± 3	39.2 ± 1.0
Day 3	139 ± 22^#^	91 ± 4	39.0 ± 1.2
Surgery	107 ± 25*	99 ± 2***	37.5 ± 1.3*

Awake recordings lasted between 30 and 45 min (each minute as a data point during recording). Values on day three of awake recordings were considered as baseline levels for the comparison with the surgical condition.

*/*** Significant difference (surgery vs. Day 3) with *p* < 0.05/0.001. ^#^ Significant difference (Day 1 and Day 2 vs. Day 3) with *p* < 0.05.

In this study, awake monitoring while being restrained is defined as a novel, non-natural, and stressful procedure (Pakkala et al., 2013). Therefore, data from day three, when birds were hypothesized to be habituated to the process and the handling person, are considered as the closest estimation for awake resting parameters of the homing pigeon and used for the comparisons with vital parameters from anesthetized pigeons.

### Anesthesia is depressing cardiopulmonary physiology, but ensures surgical tolerance

**FIGURE 3:**
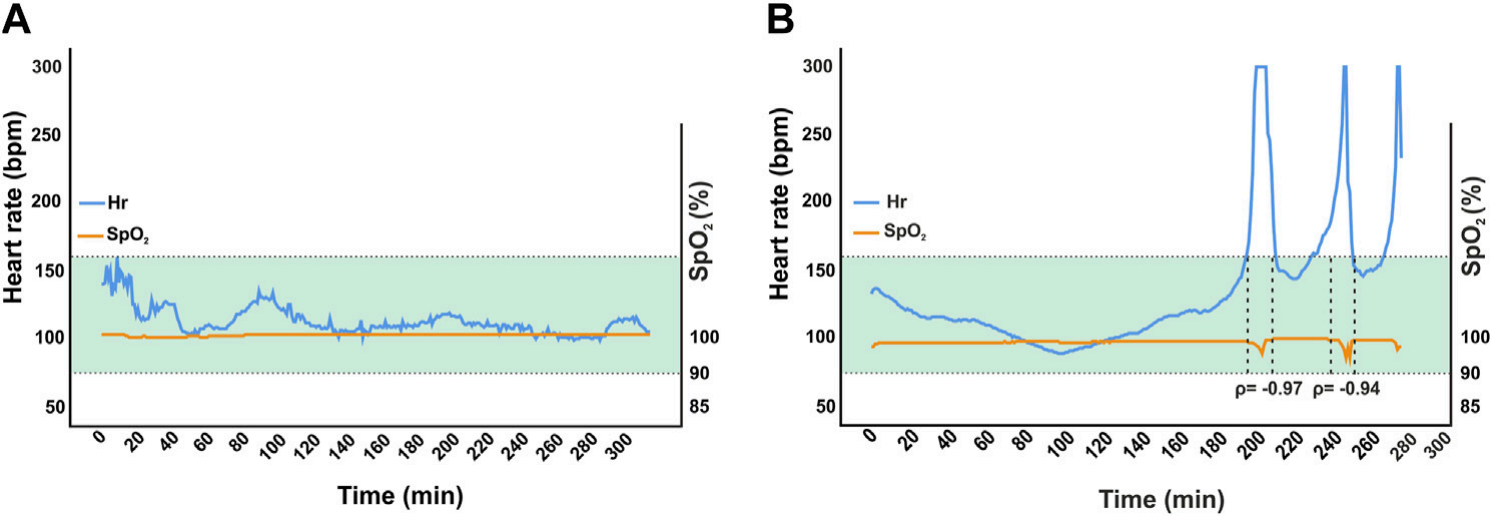
Comparison of one successful surgery **(A)** to the aborted intervention in one animal **(B)**. Mean heart rate was 110 bpm ± 10 in the former and 135 bpm ± 47 in the latter. The green area marks ± 2 standard deviations from overall mean for heart rates (*n* = 18). Induction and maintenance were comparable until the first tachycardic phase occurred in B after approximately 204 min. In this episode, heart rate exceeded 300 bpm. Therefore, anesthetic gas supply was interrupted and the animal was only supplied with 100% high flow (up to 5 L/min) oxygen. Oxygen saturation was 99%–100% in both surgeries except for occurrence of tachyarrhythmia in aborted case. Desaturation down to 92% and 86% were observed with a delay of approximately 30 s. Correlation between heart rate and oxygen saturation were significant at *ρ* = 0.033 and *ρ* = 0.017. Hemodynamic instability was assumed.

To assess the safety and efficacy of the anesthesia protocol, all 21 adult homing pigeons that underwent surgery were monitored and vital signs were recorded. For 20 pigeons, the surgery was completed as planned without incident. In one pigeon, tachyarrhythmia with heart frequencies above 300 bpm occurred during the procedure accompanied by decreasing oxygen saturation via pulse oximetry (Figures 3A, B). For this animal, following our definition of vital endangering anomalies specified in the protocol, the supply of anesthetic gas was stopped, and surgery was aborted, since these episodes of tachycardia persisted under general anesthesia and hemodynamic stability could not be ensured (Figure 1). Stopping the treatment with the anesthetic agents led to the awakening of the animal, along with complete recovery. As this procedure was interrupted and stopped, data from this session was excluded from the following analyses but will be discussed in more detail later.

Regarding cases where surgery was successfully completed, anesthesia/surgery lasted between 73 and 367 min. All animals were vitally stable during induction and evaluated to be in surgical tolerance after 15 min (Table 2; Figure 2H). After the application of anesthetic drugs, the reflex state (RS) was examined after 5, 10, and 15 min to verify the sufficiency of anesthesia induction (Figure 2H; Supplementary Table 2). The end of the anesthetic gas supply marked the beginning of the recovery period. The mean heart rate during surgery was 107 bpm ± 25. This was significantly lower compared to awake animals (t_(27)_ = −3.259, *p* = 0.003). Oxygen saturation under supply of anesthetic gas in 100% oxygen increased to 99% ±2 in anesthetized animals, which was also a significant elevation compared to awake data (U = 3.000, Z = −4.101, *p* < 0.001). Body temperature was significantly lower in anesthetized animals (37.5°C ± 1.3°C compared to 39.0°C ± 1.2 SD) despite artificial maintaining with a heating device during anesthesia to compensate for heat loss due to inhalation anesthesia but still at stable levels (t_(27)_ = −2.822, *p* = 0.009, Figures 2D–F). Exacerbation of anesthesia depth such as severe respiratory depression or hemodynamically relevant bradycardia was not observed in this study.

**TABLE 2:** Comparison of vital signs between surgeries with moderate and extended duration (mean ± SD).

	Moderate duration (*n* = 10)	Extended duration (*n* = 10)
Duration (min)	143 ± 57	291 ± 55
Heart rate (bpm)	103 ± 22	112 ± 29
SpO_2_ (%)	99 ± 1	99 ± 2
Temperature (°C)	37.4 ± 1.6	37.6 ± 1.0

The mean heart rate during drilling and implantation of recording devices (*n* = 9) was calculated separately and compared to time intervals in which these stimuli did not occur. With multiple incisions of the dura mater necessary for electrode implantation, this procedure is composed of multiple potential pain stimuli in case of insufficient anesthesia. The time window of potential painful stimuli was defined as from the first minute of drilling and bone removal to the last incision of the meninges or at least ten minutes. Under these conditions, heart rates barely deviated from mean values for intervals of moderate/low stress (t_(8)_ = 0.870, *p* = 0.410, Figure 2G).

### Balanced anesthesia ensures stability during surgeries of extended duration

**FIGURE 4:**
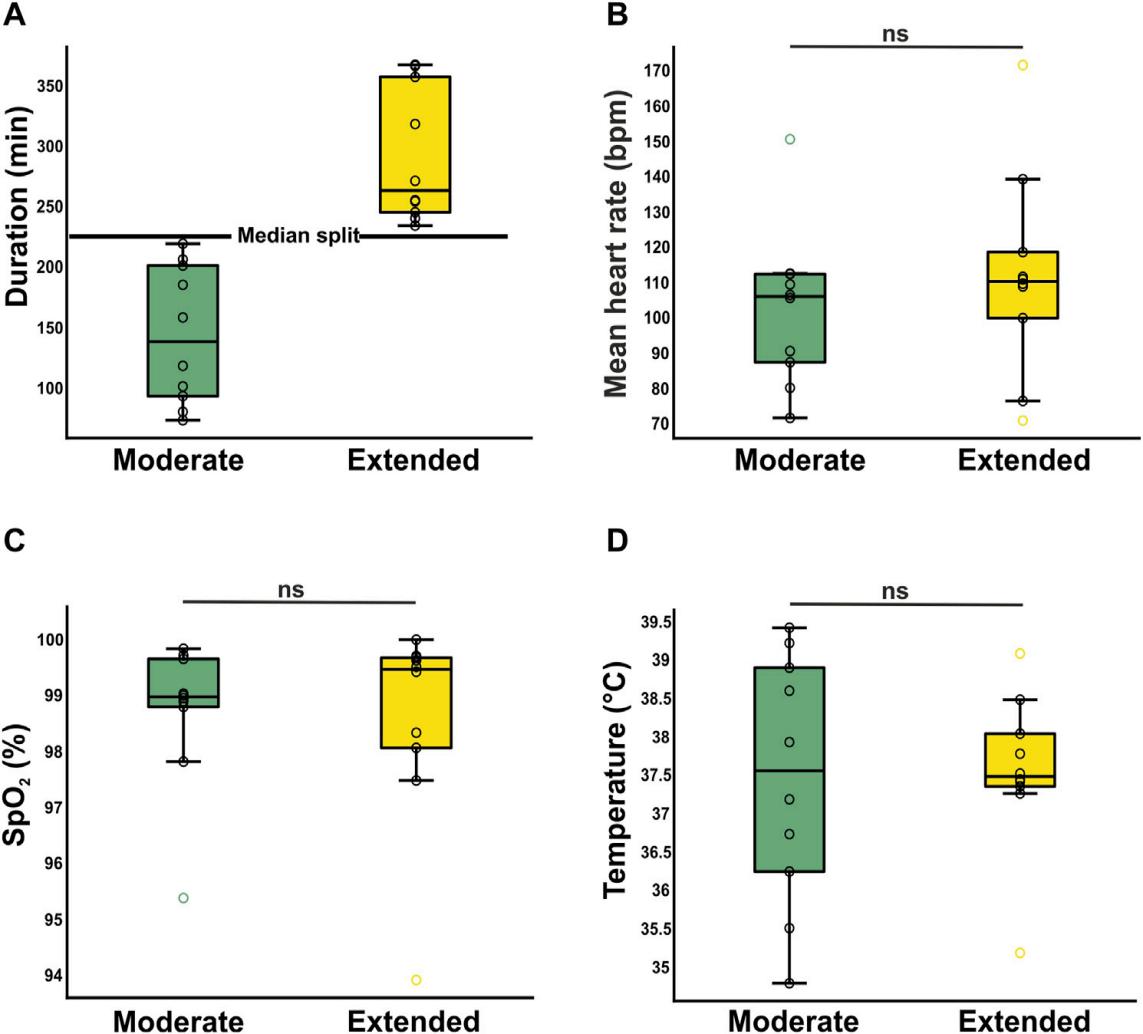
Investigation of time-dependent effects on stability of anesthesia. **(A)** Segregation into two clusters of duration [moderate (*n* = 10) and extended (*n* = 10). Both means reveal surgery/anesthesia times, which are longer than most reported studies. (B, C, D) Time dependent dynamics of vital parameters heart rate **(B)**, oxygen saturation **(C)**, and body temperature **(D)**. Mean ± SD. Boxplots represent the lower quartile (Q1), the median and the upper quartile (Q3). Whiskers represent Q1–1.5 * IQR and Q3 + 1.5 IQR.

To assess time-dependent effects of anesthesia, the 20 surgeries were divided into 10 surgeries of moderate duration and 10 surgeries of extended duration utilizing a median split. The mean duration of the moderate (143 ± 57 min) and long surgeries (291 ± 57 min) were significantly different (Figure 4A). The duration of the surgery had no significant effect on vital parameters such as heart rate (moderate: 103 ± 22 bpm/extended: 112 ± 29 bpm, t_(18)_ = −0.792, *p* = 0.439, Figure 4B), oxygen levels SpO_2_ (99% ± 1%/99% ± 2%, U = 48 Z = −0.151, *p* = 0.880, Figure 4C), or temperature (37.4°C ± 1.6°C/37.6°C ± 1.0°C, t_(18)_ = −0.172, *p* = 0.865, Figure 4D).

## Discussion

In this study, the effects of a balanced anesthesia protocol on cardiopulmonary physiology and body temperature of homing pigeons were investigated. During various surgeries typically performed in avian neuroscience, heart rate, oxygen saturation, and cloacal temperature were measured. These vital signs were compared to physiological parameters recorded in awake and habituated pigeons. This study aimed to refine established procedures for anesthesia and intraoperative analgesia in bird species and to provide a safe and reliable protocol to the neuroscience and veterinary community.

In awake recordings, we observed that the heart rate on the third day of consecutive measurements showed a significant decline compared to the first and second day of testing, most likely corresponding to habituation effects. In contrast, oxygen saturation and body temperature were not affected by repeated measurements. Earlier reported values regarding awake vital parameters of pigeons are heterogeneous. For example, the heart rates of 139 bpm on the third day of our habituation process are considerably lower than in studies that reported average heart rates of 276 (Omobowale et al., 2017) or 273 bpm (Lopez Murcia et al., 2005). However, these values are comparable to the results of studies where frequencies of 155 bpm and 160 bpm were reported (Calder, 1968; Botman et al., 2016b; Behroozi et al., 2020). The variation in the reported values may be explainable by an acute stress response evoked by measuring procedures and insufficient habituation to handling (Pakkala et al., 2013). Stress-induced changes in the pigeon’s physiology (for example, by handling, changing of housing or new handling persons) such as respiratory distress and hyperthermia is described in literature (Rashotte et al., 1998; Abou-Madi, 2001; Aguiar Bittencourt et al., 2015). Habituation to novel stimuli, including to the surgeon or the anesthesiologist and lab facilities, may have an positive impact on the occurrence of perioperative cardiac complications due to arrhythmogenicity of arousal and high stress levels (Erhardt et al., 2012). In addition, the study of Omobowale et al. (2017) is an electrocardiographic (ECG) study, where several electrodes were attached to the torso of the animal. Immobilization and extensive, unknown procedures may have been a more salient stressor than pulse oximetry. The habituation of the pigeons towards handling persons, or measurements, was not reported in the abovementioned studies. However, it has been shown, that habituation to experimental procedures leads to a significant reduction of corticosterone levels as well as heart rate in pigeons (Behroozi et al., 2020). Our findings underline the necessity to habituate birds to awake measurements to improve reproducibility and homogeneity of bird vital parameters as well as to habituate birds to preparatory procedures before surgery to reduce potential stress and its risks (Erhardt et al., 2012; Pakkala et al., 2013).

When compared to parameters of awake pigeons, intraoperative values for heart rate were significantly lower on average. The decline was an expected consequence of balanced anesthesia, as decreased heart rates are common in studies using volatile anesthetics, and injected agents such as alpha-agonists and opiates (Greene and Thurmon, 1988; Rosow, 1988; Atalan et al., 2002; Commiskey et al., 2005; Seok et al., 2016). Ketamine on the other hand opposed to previously mentioned anesthetics in terms of cardiovascular depression as dissociative anesthesia includes stimulation of the sympathetic nervous system. One of the aims was also not to choose a dose higher than necessary in order to keep neurotoxic effects as low as possible (Morris et al., 2009; Paule et al., 2011; Trimmel et al., 2018). We proposed a mutual decrease of these opposing effects and in addition, significant reduction of side effects of each administered anesthetic agent per time compared to other studies with similar dosages of each component, but significantly shorter anesthesia times (Lumeij and Deenik, 2003; Durrani et al., 2008; Botman et al., 2016b; Rehman et al., 2020). Nonetheless, our protocol resulted in stable cardiovascular physiology.

During surgical procedures, we found a significant increase in oxygen saturation in anesthetized birds, compared to awake animals due to the supplementation of oxygen alongside the volatile anesthetic. The induced elevation of the inspiratory oxygen fraction (FiO_2_) and consecutively arterial oxygen partial pressure (p_a_O_2_) therefore improves oxygen supply. Prolongation of potential apnea time and avoidance of hypoxic damage to the tissue of organs are the rationale behind it.

Furthermore, we observed a significant decrease in body temperatures of awake and anesthetized animals. Anesthetic drugs are known to alter thermoregulation which can lead to hypothermia, a common cause of post-anesthesia complications and fatalities (Rashotte et al., 1998). As small animals are quite susceptible to loss of body heat due to the relatively large surface area to volume ratio, the animals were placed on a heating pad to counteract this heat loss. While the recorded temperature of the animals undergoing surgery was lower than that of awake animals, this difference appeared to be within physiological ranges (Botman et al., 2016b; Behroozi et al., 2020; Rehman et al., 2020), indicating that using an adjustable heating pad is a simple, yet effective way of maintaining body temperature and preventing hypothermia, even in long interventions under general anesthesia (Boedeker et al., 2005).

Over the course of the surgical procedure vital signs stabilized at currently known physiological levels and showed less scattering. That was therefore interpreted as a further indication for sufficient depth of anesthesia in addition to the evaluated reflex score.

To assess the effectiveness and stability of our surgery protocol, we furthermore investigated heart rate during phases of potentially high pain salience, as well as time-dependent effects of anesthesia. Phases of potentially high pain salience were defined as starting from the first removal of the bone for the craniotomies up to the last incision of the meninges. The comparison of the heart rate in this timeframe to the rest of the surgery revealed no significant difference or any dynamics. This indicates a strong analgesic effect of our anesthesia regiment and therefore sufficient reach of surgical tolerance, ensured by ketamine, butorphanol, and isoflurane (Finck and Ngai, 1982; Miller and Buttrick, 1999; Grimm et al., 2000).

We further assessed the time dependent effects of our anesthesia protocol by comparing the vital parameters of surgeries of moderate and surgeries of extended duration. The surgery duration had no significant effect on any of the investigated vital parameters. This is essential for proposing that further administration of anesthetic drugs remains an option but may not be necessary for interventions comparable to these in our study.

In one intervention, paroxysmal tachycardia was observed, which declined after an immediate reduction of the anesthetic gas supply followed by re-induction of anesthesia with a lower dose of isoflurane. Since tachycardia reemerged three times, the procedure was stopped and the animal was supplied with oxygen in accordance with our end-point criteria. The pigeon recovered just as quickly as the other animals without noticeable impairments or other negative consequences. In this specific case, the drastic increase in heart rate was accompanied by a decrease in oxygen saturation with a delay of a few seconds. Therefore, it can be assumed that the lack of capillary perfusion caused by vasoplegic hypotension may be affecting peripheral pulse oximetry (Berridge, 1992; Shamir et al., 1999). Therefore, there is room for further research towards this aspect, especially when considering that severe vasodilatation is known to be an adverse effect of isoflurane and other volatile anesthetics of its class (Stadnicka et al., 1993; Matta et al., 1999; Akata, 2007; Noel-Morgan and Muir, 2018). The typical comparison between this intervention and a surgery without any complications outlines the special aspects of this isolated case.

Our protocol was verified by clinical outcome and stability of vital parameters within physiological ranges. Each animal survived surgery and, in one case defined physiological endpoints determined the early termination of the intervention. The conservative selection of physiological endpoints and the fact that close monitoring took place ensured adequate and early detection of hazards (McMillan and Darcy, 2016).

In our study, the focus was on minimally invasive neurosurgical procedures with a proof of concept for a safe but effective anesthetic protocol. Useful additional tools for the future study of avian physiology under different anesthesia regiments would be the implementation of invasive hemodynamic monitoring combined with non-invasive blood pressure measurements as a more direct indicator for cardiovascular physiology, which is already established in veterinary surgery and monitoring (Gunkel and La Fortune, 2005; Lichtenberger, 2005; Acierno et al., 2008). A comparison of spontaneous breathing mask ventilation and complete mechanical ventilation via an endotracheal tube for the intervention of these durations remains a topic for future investigation. This is especially relevant for small birds, since it allows capnography as an additional diagnostic and monitoring tool. In veterinary medicine, mask ventilation is commonly used (Lierz and Korbel, 2012) but intubation becomes increasingly recommended for birds (Gunkel and La Fortune, 2005).

In conclusion, in this study, we refined established surgical procedures for general anesthesia in birds. Practicability was one of our priorities, and for this reason the protocol was kept simple for researchers without veterinary backgrounds. Thus, intuitive handling is a characteristic of this anesthesia regimen. By demonstrating that vital parameters remained stable during anesthesia we confirmed that this approach ensures the birds’ safety. Our approach therefore offers a validated protocol that can be used by neuroscientists and veterinarians for prolonged surgery in birds.

## Data Availability

The raw data supporting the conclusion of this article will be made available by the authors, without undue reservation.
