# Supplementary material for: Balanced anesthesia in pigeons (Columba livia): a protocol that ensures stable vital parameters and feasibility during long surgeries in cognitive neuroscience
Source: Front Physiol. 2024 Aug 1;15:1437890. doi: 10.3389/fphys.2024.1437890 (PMC11324599; doi:10.3389/fphys.2024.1437890)
Supplement: Supplementary file 1 [file Table1.docx]

Supplementary table 1. Overview of pharmaceutical products and medical technical devices used in this study.

Pharmaceuticals		
**Name**	**Dose**	**Company**
**Butorphanole**	1.5 – 3 mg/kg bodyweight IM	Dolorex®, MSD Animal Health, Germany
**Carprofen**	1 mg/kg bodyweight IM	Rimadyl®, Pfizer Deutschland GmbH, Germany
**Isoflurane**	1-5 % in pure oxygen	Forene®, AbbVie AG, Switzerland
**Ketamine**	40 - 60 mg/kg bodyweight IM	Ketavet®, Zoetis Deutschland GmbH, Germany
**Oxygen**	0.3 - 1.5 l/min	
**Xylazine**	1 - 2 mg/kg bodyweight IM	Rompun®, Bayer AG, Germany
**Xylocaine 2 %**	0.5 - 1.5 ml local application	AstraZeneca GmbH, Germany
**Apparatus**		
**Purpose**	**Trade name**	**Company**
**Monitor**	Datalys V5 Veterinary Monitor	Lutech veterinary Industries Inc., NY, US
**Pulse Oximeter**	Nellcor Y sensor and lingual clip.	Lutech veterinary Industries Inc., NY, US
**Thermometer**	Vet Rectal Temperature Sensor	Lutech veterinary Industries Inc., NY, US
**Vaporizer / anaesthetic machine**	Komesaroff Mark V	Medical development international limited, Australia
