# Supplementary material for: Balanced anesthesia in pigeons (Columba livia): a protocol that ensures stable vital parameters and feasibility during long surgeries in cognitive neuroscience
Source: Front Physiol. 2024 Aug 1;15:1437890. doi: 10.3389/fphys.2024.1437890 (PMC11324599; doi:10.3389/fphys.2024.1437890)
Supplement: Supplementary file 2 [file Table2.docx]

Supplementary table 2. Reflex score for evaluation of anaesthesia depth for birds. A maximum of 3-4 points is considered as surgical tolerance accompanied with absence of vigilance and nociception. This is a shortened scheme inspired by Korbel et al.(Erhardt et al., 2012). Evaluation is scheduled after 5, 10 and 15 minutes.

Evaluation of anaesthesia depth: Score sheet			
	5 min	10 min	15 min
**Vigilance *<br>Awake = 2<br>Soporific = 1<br>Asleep = 0**			
**Head position<br>Hanging without muscle tonicity = 0<br>Slight to significant head raise = 1**			
**Neck tonicity<br>Yes = 1<br>No = 0**			
**Leg tonicity<br>Yes = 1<br>No = 0**			
**Corneal reflex (Touch of the nictitating membrane with a cotton swab)<br>No reflex = 0<br>Delayed, incomplete movement of the nictitating membrane = 1<br>Delayed, complete movement of the nictitating membrane = 2<br>Physiological reflex = 3**			
**Pectoralis reflex (Pinching the skin between the ossa pubis with a forceps)<br>No reflex = 0<br>Slight wing movement = 1<br>Tightening of some body parts = 2<br>Massive defensive movements = 3**			
**Interphalangeal reflex (Pinching of interdigital skin)<br>No reflex = 0<br>Slight leg tightening = 1<br>Significant leg tightening/ eye movements = 2**			
**Cloacal reflex (Pinching the pericloacal skin)<br>No reflex = 0<br>Contraction of cloacal sphincter muscle = 1<br>Tightening of body parts/ head movements = 2<br>Massive defensive movements = 3**			

* Unconsciousness and lack of response to pain stimuli are mandatory prerequisites for determining the score.
